# Mitochondrial Proteins Unveil the Mechanism by Which Physical Exercise Ameliorates Memory, Learning and Motor Activity in Hypoxic Ischemic Encephalopathy Rat Model

**DOI:** 10.3390/ijms23084235

**Published:** 2022-04-11

**Authors:** Fred Gendi, Feifei Pei, Yuan Wang, Haoye Li, Jia Fu, Cheng Chang

**Affiliations:** School of Basic Medical Sciences, Zhengzhou University, Zhengzhou 450000, China; genddyfred@gmail.com (F.G.); peifeifei950815@gmail.com (F.P.); wy2818906221@gs.zzu.edu.cn (Y.W.); 202012402015805@gs.zzu.edu.cn (H.L.); monkeyking3111@gmail.com (J.F.)

**Keywords:** exercise, apoptosis-inducing factor, mitochondria, hypoxia, ischemia

## Abstract

Background: Physical exercise has been shown to improve cognitive and motor functions, promoting neurogenesis and demonstrating therapeutic benefits in neurodegenerative disorders. Nonetheless, it is crucial to investigate the cellular and molecular mechanisms by which this occurs. The study aimed to investigate and evaluate the effect of swimming exercise on the changes of mitochondrial proteins in the brains of rats with hypoxic ischemic encephalopathy (HIE). Methods: the vertical pole and Morris water maze tests were used to assess the animals’ motor and cognitive functions, and western blot and immunofluorescence of brain tissue were used to assess the biomarkers of mitochondrial apoptosis and cristae stability in response to exercise training. Four groups of rats were used: (1) sham sedentary group (SHAM, NT), (2) sham exercise training group (SHAM, T) (3) hypoxic ischemic encephalopathy sedentary group (HIE, NT), and (4) hypoxic ischemic encephalopathy exercise training group (HIE, T). Results: animals with HIE showed motor and cognitive deficits, as well as increased apoptotic protein expression. Exercise, on the other hand, improved motor and cognitive functions while also suppressing the expression of apoptotic proteins. Conclusions: By stabilizing the mitochondrial cristae and suppressing the apoptotic cascade, physical exercise provided neuroprotection in hypoxic ischemia-induced brain injury.

## 1. Introduction

Hypoxic-ischemic encephalopathy (HIE) is a type of serious central nervous system damage in neonates caused by perinatal hypoxia. HIE is a primary cause of infant death in both term and preterm neonates, as well as serious chronic problems, including cerebral palsy, neurosensory abnormalities, and cognitive deficits [1,2]. HIE affects 1 to 8 out of 1000 live births in developed nations and up to 26 out of 1000 live births in developing countries, accounting for up to 23% of all infant deaths worldwide [3,4].

Perinatal abnormalities, such as hypoxia, can cause brain damage, which is commonly followed by neurological disorders, such as cerebral palsy or mental retardation. The processes driving newborn brain damage remain unclear; however, mitochondria play a critical role, not only are they essential for metabolism, but they also contain several proteins with apoptotic capabilities [5]. Exercise reduces brain injury-induced motor deficits by suppressing apoptotic neuronal cell death in the motor cortex [6]. Moreover, it has gained a reputation among pediatric physical therapists as an intervention of choice for children with cerebral palsy [7]. With accruing evidence from animal and human studies supporting the view that physical exercise enhances neuroplasticity and thus reduces the risk of several neurodegenerative diseases [8,9,10]. While many studies [11,12,13,14] concur that physical exercise improves brain function by promoting neuroplasticity and cognition, few have sought to elucidate the response of mitochondrial proteins involved in apoptosis to physical exercise. With exercise being so important for maintaining good health when done regularly, a better understanding of the molecular mechanisms by which exercise affords beneficial effects to the body is fundamental to identifying more specific pathways that can be manipulated to prevent or treat diseases [15]. Mitochondria play an important role in neonatal neurodegeneration following hypoxia stress, and their dysfunction is a critical stage in neurodegenerative progression, which is associated with subsequent induction of cell death pathways that is a key hallmark of hypoxic-ischemic injury [16]. Mitochondria activate several apoptotic signaling pathways and protein interactions, including; cytochrome c (Cyto.C), apoptosis-inducing factor (AIF), endonuclease G (Endo G), and second mitochondria-derived activator of caspase (Smac), to discharge pro-apoptotic proteins from the intermembranes, culminating in intrinsic apoptosis [17,18]. The mitochondrial pathway, which signals cell death by apoptosis, is activated by a variety of stressors, including hypoxic ischemia. The permeabilization of the outer mitochondrial membrane, OMM [also referred to as mitochondrial outer membrane permeabilization (MOMP)] is the primary event in mitochondrial mediated apoptosis, allowing different mitochondrial proteins to relocate to the cytosol and enhancing procaspase activation [19]. Furthermore, [20] reported that exercise increases the copy number of mitochondrial DNA (mtDNA) in the cortex and hippocampus, which is directly affected in HIE.

This study examined the effects of exercise on cognitive and motor performance by highlighting the response of mitochondrial apoptotic proteins following hypoxic ischemic insult and exercise. We investigated the effects of swimming exercise on mitochondrial apoptosis in the hippocampus and cerebral cortex in HIE-induced motor and cognitive impairment.

## 2. Results

### 2.1. Swimming Exercise Promotes Motor and Cognitive Performance

**Figure 1:**
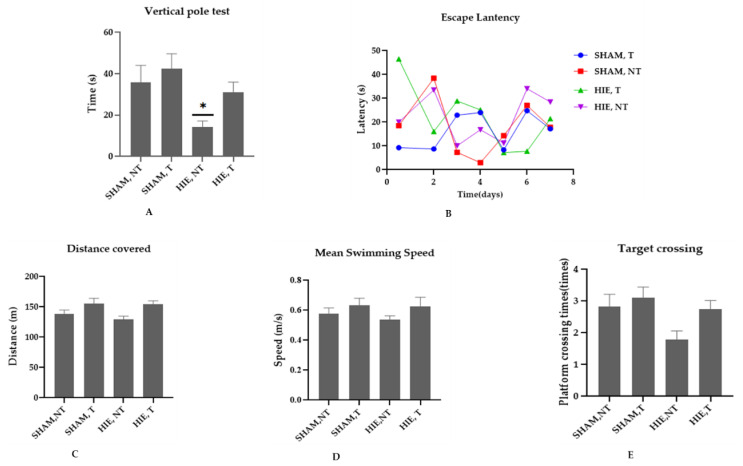
Effect of swimming on motor and cognitive performance. (**A**) Vertical pole test; exercise training animals (SHAM, T and HIE, T) spent longer time on the vertical pole than sedentary animals (SHAM, NT and HIE, NT), with HIE, NT showing a statistically significant decrease; findings are expressed as Mean ± SEM, * *p* < 0.05 (one-way ANOVA followed by Dunnett’s post-test). (**B**) Escape latency; swimming exercise enhanced learning and memory; exercising animals showed a significantly reduced escape latency, or the time (seconds) required to escape. (**C**) Distance covered; in the exercising groups, there was an obvious increase in distance covered, but it was not statistically significant at *p* < 0.05 (one-way ANOVA followed by Dunnett’s post-test). (**D**) Mean swimming speed; animal groups that were subjected to exercise showed a higher swimming speed compared to the sedentary groups indicating that their motor behavior was positively influenced by exercise. (**E**) Target crossing; this shows the number of times the animals crossed the site at which the platform had been positioned which as expected improved with exercise. The results are expressed as Mean ± SEM. The number of animals used in each group is 7, but the difference was not statistically significant at *p* < 0.05 (one-way ANOVA followed by Dunnett’s post-test).

We conducted the vertical pole and Morris water maze (MWM) tests to determine whether swimming exercise improves motor, learning, and spatial memory performance in HIE rats. According to [21], the vertical pole test is used to assess motor performance, and the results in Figure 1A showed that the motor performance of the HIE rats was lower than that of the control rats (*p* < 0.05). Swimming exercise, on the other hand, improved the motor performance of rats, though not statistically significantly at (*p* < 0.05). The MWM is a spatial learning task that requires rats to locate a hidden platform in an opaque pool of water using visual cues. Acquisition of spatial learning in both control rats and HIE rats was observed as reduced latency to reach the hidden platform by day 6. Rats that undertook swimming exercise significantly outperformed the control rats as shown in Figure 1B. To evaluate the reference memory, we conducted a probing test 24 h after the final training session (day 6), which was conducted without the hidden platform. As expected, exercising rats demonstrated considerable memory enhancement, as shown by the increased number of times they crossed the target quadrant (Figure 1E). During the MWM sessions, the control and exercise training rats traveled varied distances and swam at varying speeds (Figure 1C,D). These findings suggest that swimming exercise improves motor and cognitive function. Taken together, our results support the concept that exercise improves motor function, spatial learning, and memory retention.

### 2.2. Mitochondrial Proteins in the Cytoplasm of the Hippocampus

**Figure 2:**
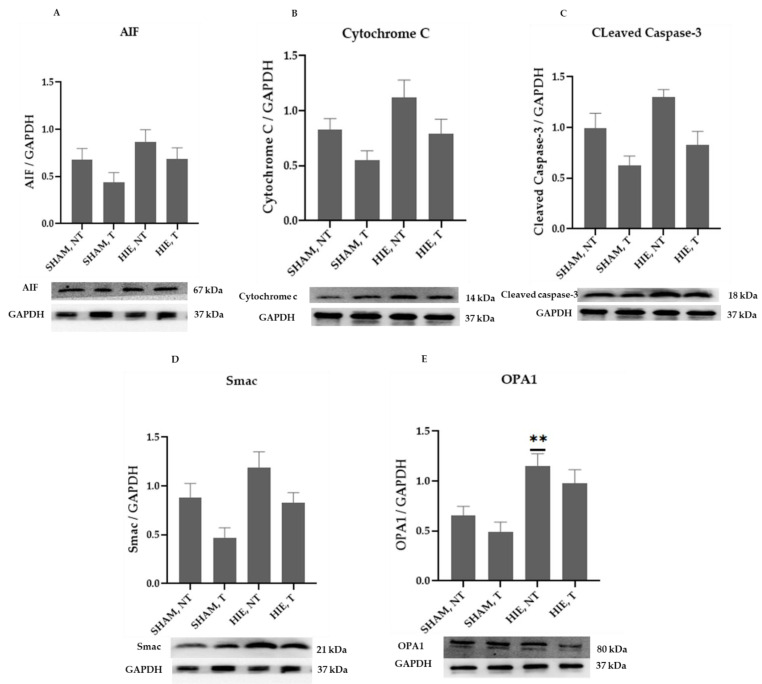
Protein changes in the cytoplasm of the hippocampus. Examples of Western blot bands normalized to GAPDH are presented (with the matching wells). Units are presented relative to GAPDH content. (**A**) AIF, apoptosis-inducing factor, (**B**) cytochrome c, (**C**) cleaved caspase-3, (**D**) Smac/Diablo, and (**E**) OPA1. Asterisks denote statistically significant differences between groups (*, vs. SHAM, NT). One asterisk (*) denotes *p* < 0.05 (**) denote *p* < 0.01. F-values after running one-way ANOVAs with Dunnett’s post hoc multiple comparison test were: F = 2.264 (AIF), F = 3.775 (cytochrome c), F = 6.125 (cleaved caspase-3), F = 5.140 (Smac/Diablo) and F = 7.096 (OPA1). HIE, NT denotes animals with hypoxic ischemic encephalopathy that did not undergo exercise training; HIE, T denotes animals with hypoxic ischemic encephalopathy that received exercise training; SHAM, NT denotes normal animals that did not receive exercise training; and SHAM, T denotes normal animals that received exercise training.

The mitochondrial apoptosis indicators in the cytosolic portion; cytochrome c, apoptosis-inducing factor (AIF), and cleaved caspase-3, as well as Smac/Diablo and OPA1, increased appreciably in HIE, as demonstrated by Western blot semi-quantification (Figure 2). Cytochrome c (Figure 2B), cleaved caspase-3 (Figure 2C), AIF (Figure 2A), and Smac/Diablo (Figure 2D) were all expressed differently in HIE with and without exercising, but there was no statistically significant difference when compared to SHAM, NT. Nevertheless, cytosolic OPA1 showed a statistically significant difference in HIE, NT, Figure 2E.

### 2.3. Mitochondrial Proteins in the Nuclei of the Hippocampus

**Figure 3:**
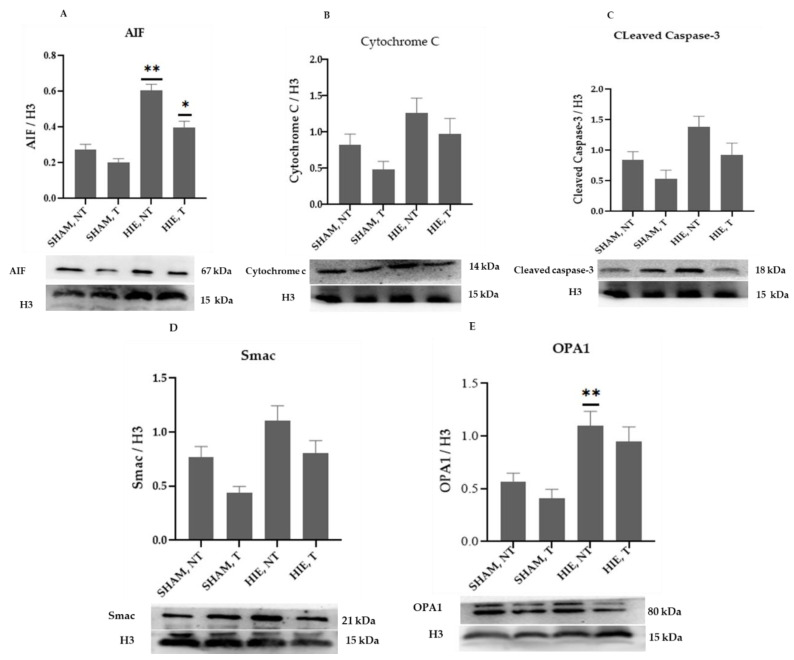
Protein changes in the nuclei of the hippocampus. Examples of Western blot bands normalized to histone 3 (H3) are presented (with the matching wells). Units are presented relative to H3 content. (**A**) AIF, apoptosis-inducing factor, (**B**) cytochrome c, (**C**) cleaved caspase-3, (**D**) Smac/Diablo, and (**E**) OPA1. Asterisks denote statistically significant differences between groups (*, vs. SHAM, NT). One asterisk (*) denotes *p* < 0.05, (**) denote *p* < 0.01 F values were; F = 32.870 (AIF), F = 3.510 (cytochrome c), F = 4.733 (cleaved caspase 3), F = 6.650 (Smac/Diablo) and F = 7.896 (OPA1). HIE, NT denotes animals with hypoxic ischemic encephalopathy that did not undergo exercise training; HIE, T denotes animals with hypoxic ischemic encephalopathy that were subjected to exercise training; SHAM, NT denotes normal animals that did not receive exercise training, and SHAM, T denotes normal animals that received exercise training.

The mitochondrial apoptosis indicators in the nuclear portion, cytochrome c, apoptosis-inducing factor (AIF), and cleaved caspase-3, as well as Smac/Diablo and OPA1, increased significantly in HIE, as demonstrated by Western blot semi-quantification (Figure 3). Cytochrome c (Figure 3B), cleaved caspase-3 (Figure 3C), and Smac/Diablo (Figure 3D) were all expressed differently in HIE with and without exercise but there was no statistically significant difference when compared to SHAM, NT. Nonetheless, nuclear AIF and OPA1 (Figure 3A,E) showed statistically significant changes.

### 2.4. Mitochondrial Proteins in the Cytoplasm of the Cerebral Cortex

**Figure 4:**
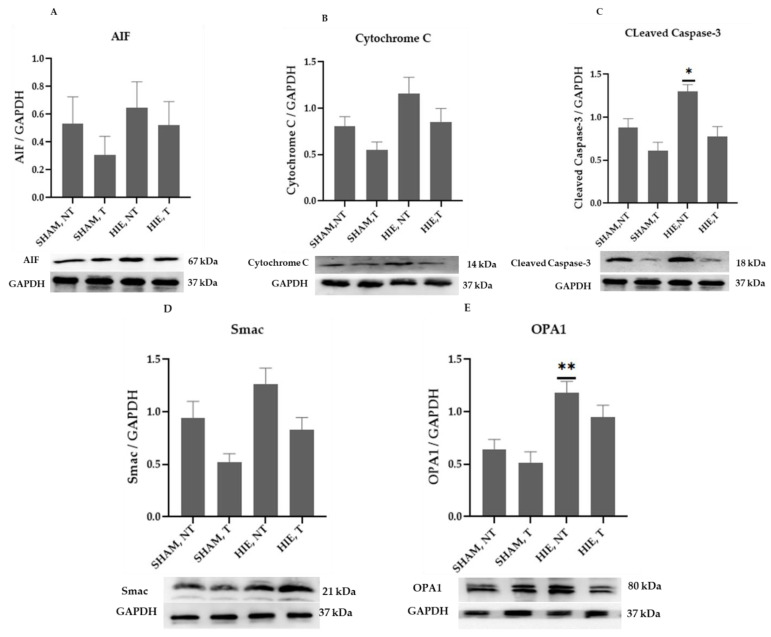
Protein changes in the cytoplasm of the cerebral cortex. Examples of Western blot bands normalized to GAPDH are presented (with the matching wells). Units are presented relative to GAPDH content. (**A**) AIF, apoptosis-inducing factor, (**B**) cytochrome c, (**C**) cleaved caspase-3, (**D**) Smac/Diablo, and (**E**) OPA1. Asterisks denote statistically significant differences between groups (*, vs. SHAM, NT). One asterisk (*) denotes *p* < 0.05, (**) denote *p* < 0.01.F values were; F = 0.6974 (AIF), F = 3.666 (cytochrome c), F = 8.992 (cleaved caspase 3), F = 5.643 (Smac/Diablo), and F = 8.257 (OPA1). HIE, NT denotes animals with hypoxic ischemic encephalopathy that did not receive exercise training, HIE, T denotes animals with hypoxic ischemic encephalopathy that received exercise training; SHAM, NT denotes normal animals that did not undergo exercise training; and SHAM, T denotes normal animals that received exercise training.

The mitochondrial apoptosis indicators in the cytoplasmic portion, apoptosis-inducing factor (AIF), cytochrome c, cleaved caspase-3, as well as Smac/Diablo and OPA1, increased significantly in HIE, as demonstrated by Western blot semi-quantification (Figure 4). AIF (Figure 4A), Cytochrome c (Figure 4B), and Smac/Diablo (Figure 4D) were all expressed differently in HIE with and without exercise but there was no statistically significant difference when compared to SHAM, NT. Despite this, cleaved caspase-3 (Figure 4C) and OPA1 (Figure 4E) showed statistically significant changes.

### 2.5. Mitochondrial Proteins in the Nuclei of the Cerebral Cortex

**Figure 5:**
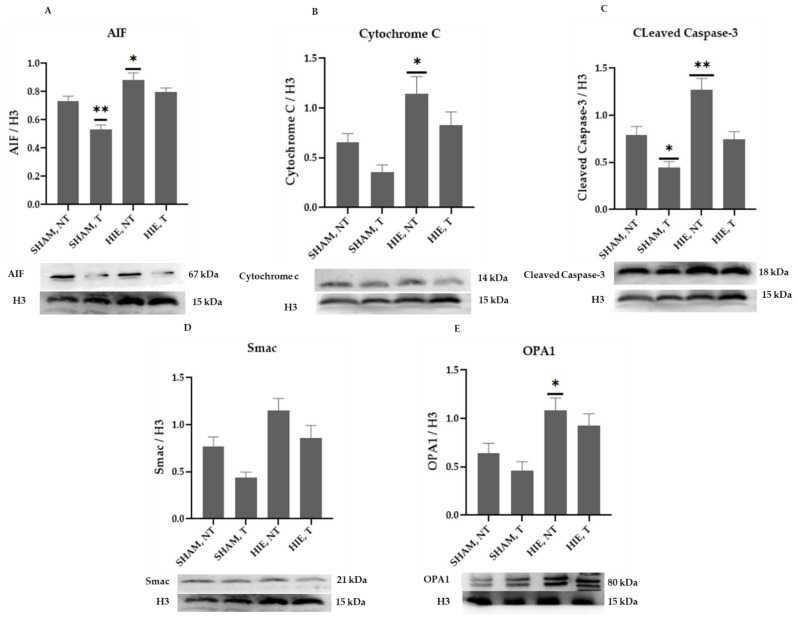
Protein changes in the nuclei of the cerebral cortex. Examples of Western blot bands normalized to H3 are presented (with the matching wells). Units are presented relative to H3 content. (**A**) AIF, apoptosis-inducing factor, (**B**) cytochrome c, (**C**) cleaved caspase-3, (**D**) Smac/Diablo, and (**E**) OPA1. Asterisks denote statistically significant differences between groups (*, vs. SHAM, NT). One asterisk denotes *p* < 0.05, two asterisk (**) denote *p* < 0.01.F values were; F = 16.03 (AIF), F = 6.991 (cytochrome c), F = 14.36 (cleaved caspase 3), F = 6.903 (Smac/Diablo) and F = 5.895 (OPA1). HIE, NT denotes animals with hypoxic ischemic encephalopathy that did not undergo exercise training; HIE, T denotes animals with hypoxic ischemic encephalopathy that underwent exercise training; SHAM, NT denotes normal animals that did not undergo exercise training; and SHAM, T denotes normal animals that received exercise training.

The mitochondrial apoptosis indicators in the nuclear portion, cytochrome c, apoptosis-inducing factor (AIF), and cleaved caspase-3, as well as Smac/Diablo and OPA1, increased significantly in HIE, as demonstrated by Western blot semi-quantification (Figure 5), AIF (Figure 5A), Cytochrome c (Figure 5B), cleaved caspase-3 (Figure 5C), and OPA1 (Figure 5E) were all expressed differently in HIE and exercise and were statistically significant compared to SHAM, NT. Changes in nuclear Smac/Diablo (Figure 5D), on the other hand, were not statistically significant.

Taken together, these findings support the proposition that exercise improves motor function, learning, and memory recovery by suppressing hippocampal and cortical mitochondrial apoptotic proteins expression in the brain of HIE rats.

### 2.6. Immunofluorescence Analysis of Proteins in the Motor Cortex

**Figure 6:**
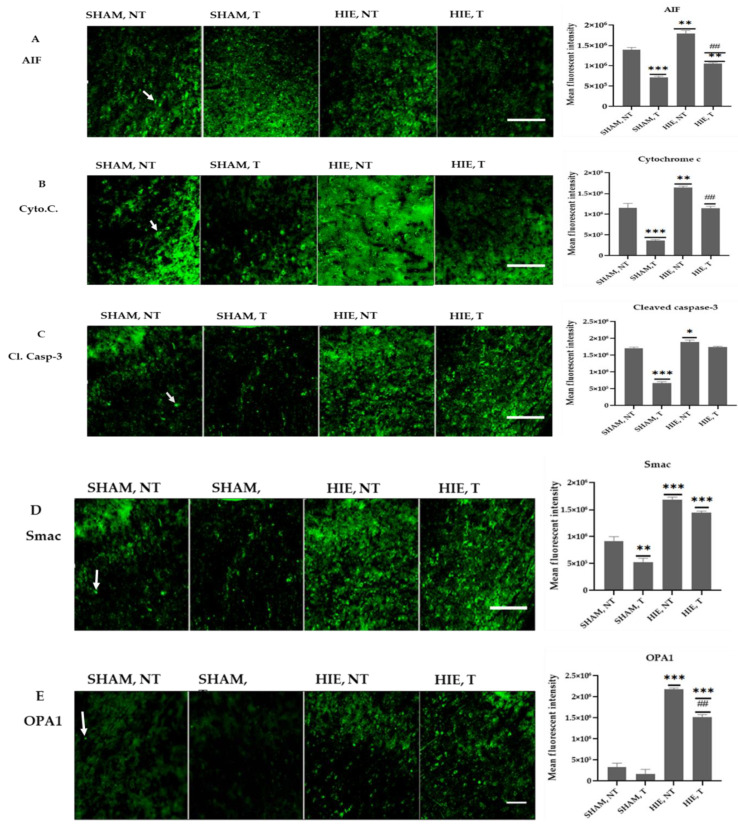
Immunofluorescence images and mean fluorescent intensities of the proteins in the motor cortex. (**A**) Representative immunofluorescence images and MFI of AIF in the motor cortex of each group. AIF molecules are green. AIF molecules were analyzed in the motor cortex. Results are presented as the Mean ± SEM of 3 rats from each group. (** *p* < 0.001 and *** *p* < 0.0001) compared with the SHAM, NT group; (^##^ *p* < 0.001 compared with the HIE, NT group; each group, *n* = 3). Scale bar, 100 µm. (**B**) Representative immunofluorescence images and MFI of Cytochrome C in the motor cortex of each group. Cytochrome C molecules (green) were analyzed in the motor cortex. Data are presented as the Mean ± SEM of 3 rats from each group. (** *p* < 0.001 and *** *p* < 0.0001) compared with the SHAM, NT group; (^##^ *p* < 0.001 compared with the HIE, NT group); each group, *n* = 3. Scale bar, 100 µm. (**C**) Representative immunofluorescence images and MFI of Cleaved caspase-3 in the motor cortex of each group. Cleaved caspase-3 molecules (green) were analyzed in the motor cortex. Results are presented as the Mean ± SEM of 3 rats from each group. (* *p* < 0.05 and *** *p* < 0.0001) compared with the SHAM, NT group); each group, *n* = 3. Scale bar, 100 µm. (**D**) Representative immunofluorescence images and MFI of SMAC in the motor cortex of each group. SMAC molecules (green) were analyzed in the motor cortex. Results are presented as the Mean ± SEM of 3 rats of each group. (** *p* < 0.001 and *** *p* < 0.0001) compared with the SHAM, NT group); each group, *n* = 3. Scale bar, 100 µm. (**E**) Representative immunofluorescence images and MFI of OPA1 in the motor cortex of each group. OPA1 molecules (green) were analyzed in the motor cortex. Individual data are presented as the Mean ± SEM from 3 rats in each group. (*** *p* < 0.0001) compared with the SHAM-NT group; (^##^ *p* < 0.001 compared with the HIE, NT group); each group, *n* = 3. Scale bar, 100 µm.

**Figure 7:**
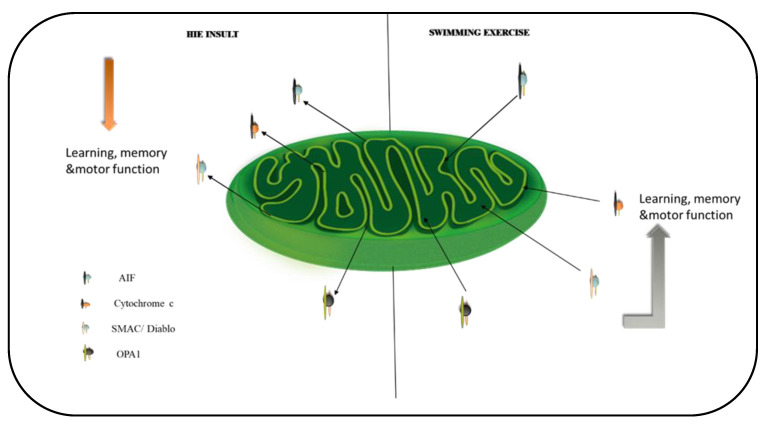
A schematic summary of the effect of exercise on mitochondrial proteins in the brain. Exercise downregulates the translocation of the proteins to the cytosol and nuclei of the hippocampus and cortex. This stabilizes the mitochondria and mediates exercise’s positive effects on learning, memory, and motor function.

Figure 6 shows representative immunofluorescence images from the *motor cortex* of the cerebral cortex region stained for AIF, Cytochrome C, Cleaved caspase-3, Smac, and OPA1. The quantitative analysis of the mean fluorescent intensity (MFI) of the proteins is also summarized in Figure 6 and Figure 7. Swimming exercise caused a significant reduction in the expression of the proteins. Images and the mean fluorescent intensity (MFI) graphs demonstrated significant changes in the expression of proteins in the motor cortex, which has been shown to control motor learning and voluntary movement [22], and the proteins showed a drastic significant reduction in exercising groups when compared to SHAM, NT (Figure 6A–E). Moreover, HIE, T motor cortex had a significant decrease in protein expression compared to HIE, NT (Figure 6A,B,E).

## 3. Discussion

Due to the fact that mitochondria are essential for energy production within brain cells [23,24], we investigated the effect of swimming exercise on mitochondrial apoptotic and dynamic signals in HIE. To accomplish this, we used an exercise routine and assessed five mitochondrial function regulators (AIF, Cytochrome c, Cleaved caspase-3, Smac/Diablo, and OPA1). Our results demonstrated that exercise training affects these proteins regardless of the health state (that is, in HIE rat models or sham groups). Swimming exercise lowered the expression of mitochondrial apoptosis-related proteins; AIF, cytochrome c, and cleaved caspase-3 in the cytosol and nuclei of the hippocampus, and cortex, which is consistent with the statement made by Moore et al. [25] that physical activity impacts every aspect of the mitochondrion. Furthermore, the four weeks of exercise lowered the expression levels of the fission marker Smac/Diablo and the cristae remodeling protein OPA1, which was concomitant with enhanced motor performance, learning, and memory retention.

In the hippocampus and cerebral cortex of HIE and sedentary rats, the levels of mitochondrial apoptotic indicators; cytochrome c, AIF, and cleaved caspase-3 increased significantly. In comparison to SHAM, NT rats, when HIE and normal rats were subjected to swimming exercise, the levels of AIF, cytochrome c, and cleaved caspase-3 decreased significantly, an observation in tandem with [24,26] findings that exercise restores mitochondria function in neurodegenerative disorders. With AIF pointed out as a major contributor to neuron loss in the immature brain following hypoxia-ischemia and a hypomorphic mutation causing decreased AIF expression reported to protect against neonatal hypoxic ischemia [27], we observe here that swimming exercise has the same effect in the HIE rat brain by suppressing the protein’s cytosolic and nuclear translocation. The antiapoptotic impact of exercise training corresponds to improvements in motor function, learning, and memory, implying that exercise training circumvents mitochondrial malfunction and apoptosis. This explains the neuronal protective mechanisms reported after exercise training that promotes neurogenesis and myelin repair in the penumbra following stroke [28], as these are high energy demanding processes that necessitate stable functional mitochondria. Given exercise’s ability to modulate mitochondrial protein stabilization, enhance motor function, learning, and memory, it would be remarkable to determine whether the exercise-related metabolite, lactate, that crosses the blood-brain barrier [29] contributes significantly to exercise’s beneficial effects of suppressing apoptosis and promoting motor function, learning, and memory in HIE.

Swimming exercise significantly improved motor activity, memory, and learning in rats with HIE by reducing mitochondrial apoptosis through the Cyto.C/Cleaved Caspase-3 and AIF signaling pathways. Aside from that, swimming exercise intervention has been shown to stabilize mitochondrial cristae and membrane potential in HIE rats, as evidenced by Smac/Diablo and OPA1 reversal in these animals, which is consistent with [30,31] findings emphasizing the importance of OPA1 in mitochondrial cristae stabilization. With the underlying neurobiological mechanisms of exercise-induced neuroplasticity still mostly elusive [8], our findings provide an intriguing and plausible platform for future research on identifying additional molecular pathways that could be modulated to effectively manage HIE and the resulting impairments.

## 4. Materials and Methods

### 4.1. Animals and Experimental Groups

**Figure 8:**
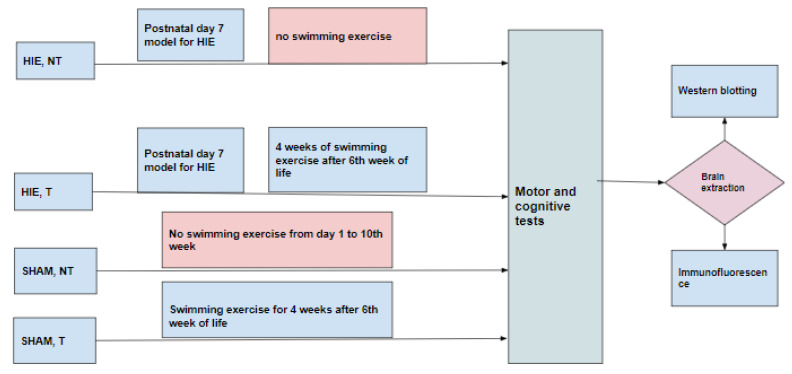
Experimental design.

Thirty-six Sprague-Dawley rats were used for this study. The animals were maintained at 22 ± 1 °C with light/dark cycles of 12 h and had free access to food and water. Experimental procedures were conducted with the approval of the Ethical Institutional Committee on Animal Care and Research of Zhengzhou University. Every effort was made to reduce the number of animals and minimize animal suffering during the experiments. Animals were randomly divided into four groups (Figure 8). (1) HIE, NT (hypoxia-ischemia encephalopathy group without exercise training) consisted of animals that were modeled for hypoxic-ischemic encephalopathy on postnatal day 7 by anesthetizing the pups with isoflurane (5% induction, 1.5% maintenance). The left common carotid artery was then permanently ligated, and the pups were put on a warm recovery couch for 30 min before they were transferred to a hypoxic chamber with a continuous flow of the hypoxic gas mixture of 92% N_2_ and 8% O_2_ for 90 min and then returned to the cages up to the 10th week without subjecting them to swimming exercise. (2) HIE, T (hypoxia-ischemia encephalopathy group with exercise training) was comprised of animals that were modeled for hypoxic-ischemic encephalopathy as in (1) and returned to the cages up to the 6th week of life and then subjected to 90 min of swimming exercise daily for five days with 2 days of rest per week for 4 weeks, (3) SHAM, NT (control group) included animals that were not subjected to any treatment but lived a normal life in the cages from postnatal day 1 to the 10th week without exercise training, and (4) SHAM, T consisted of animals not subjected to any treatment but lived a normal life in the cages from postnatal day1 up to 6 weeks, after which they were subjected to swimming exercise for 4 weeks.

After 10 weeks of life, the animals were subjected to the motor and cognitive tests (vertical pole test and Morris water maze test).

The animal brains from the different groups were then harvested and prepared for western blotting (hippocampus and cerebral cortex) and immunofluorescence (motor cortex).

### 4.2. Exercise Paradigm

Animals were housed with food and water *ad libitum* and maintained on a 12 h light/dark cycle. They were divided into two groups: sedentary animals (HIE, NT and SHAM, NT) and exercising animals (HIE, T and SHAM, T). The exercising animals were subjected to swimming exercise training after postnatal week 6 for 4 weeks; they were subjected to swimming exercise for 5 days a week with 2 days of rest.

Each swimming session lasted for 90 min. The pool was filled to a depth of 50 cm to prevent the animals from touching the bottom of the tank. The animals were allowed to freely swim, without any extra load and were gently stimulated during swimming.

The rats were carefully dried after the exercise and returned to the cages.

### 4.3. Vertical Pole Motor Test

The animals in each group were one by one placed face-up on a cloth tape-covered pole (3.0 cm diameter and 150 cm length), which was held in a horizontal position, then was gradually lifted to a vertical position, and the time the rat stayed on the pole was recorded for a maximum of 2 min (120 s). In this test, the animal with a deficit in motor coordination and balance falls off the pole.

### 4.4. Morris Water Maze Test (MWM)

Rats (10 weeks old) were subjected to MWM after undergoing 4 weeks of exercise training protocol (SHAM, T and HIE, T). All water maze data were recorded using the Panlab SMART video tracking system (Panlab Howo Biotechnology (Shanghai) Co., Ltd.). (Shanghai, China). The MWM was used as described by [32]. Briefly, rats used visual cues placed on the borders of a swimming pool to reach a hidden platform and escape from the water. Learning was assessed across 7 days. Before learning assessment, rats were introduced into the pool that contained clear water and a visible platform. During the training, the rats were given trial swimming exercise to become familiar with the task. During the learning phase, rats were smeared with black dye for easy video tracking and the platform was submerged. Each rat was subjected to four trials from different starting points (quadrants). Latency or the time required to reach the platform was recorded every day by the Panlab SMART Video Tracking System. On the last day of the experiment, the platform was removed, and each rat was reintroduced into the water and the number of times the animals crossed the site of the hidden platform in the quadrant that previously contained the platform (target quadrant) was recorded.

### 4.5. Western Blotting

Six rats were randomly selected from each group for the protein level experiment. Proteins were extracted from the hippocampus and cortex and homogenized in RIPA’s reagent (CW2333S, CoWin Biosciences, Cambridge, MA, USA) and separated into cytoplasmic and nuclear proteins. The protein concentrations were determined using the bicinchoninic acid assay (BCA) (CW0014S, CoWin Biosciences, Cambridge, MA, USA), and a total of 20 g of protein was separated by electrophoresis on universal SDS-PAGE gels (CFAS Any KD PAGE) # PE008, Zhonghui Hecai Bio-pharmaceutical Technology Co., Ltd., Shaanxi, China. Proteins were then transferred onto polyvinylidene fluoride membranes, (PVDF) (R1CB12934, Merck Millipore Ltd., (Burlington, MA, USA). The membranes were blocked for 2 h with 5% bovine serum albumin (BSA) (#A8020, Solarbio Life Sciences, Beijing, China) at room temperature and incubated with the following primary antibodies from Cell Signaling Technology (Danvers, MA, USA): AIF (D39D2, 1:1000), Cytochrome C (136F3, 1:1000), cleaved caspase-3 (Asp175, 1:1000), Smac/Diablo (D553R, 1:1000), and OPA1 (D7C1A, 1:1000). GAPDH from Servicebio, Wuhan, China (GB11002, 1:2000), and H3 from Proteintech (17168-1-AP, 1:1000) at 4 °C overnight followed by HRP-conjugated secondary antibody SA00001-2 from Proteintect (1:5000) incubation for 2 h at room temperature. The protein bands were visualized with an enhanced chemiluminescence kit #KF005 from Affinity Biosciences and imaged with the Bio-Image Analysis system (Zhengzhou University, Zhengzhou, China). The ratios of protein band intensities to GAPDH (cytoplasmic proteins) and H3 (nuclear proteins) as internal references were determined using ImageJ.

### 4.6. Immunofluorescence

The 25-µm coronal slices of brain tissue were obtained using a freezing microtome (Leica, Germany) for immunofluorescence. The slides were blocked for 2 h with PBS with 10% fetal bovine serum, (#A8020, Solarbio Life Sciences, Beijing, China) and 0.3% Triton ×-100, Amresco 0694, Biosharp, Estonia at room temperature and then incubated with primary antibodies from Cell Signaling Technology (Danvers, MA, USA): anti-AIF, D39D2 (dilution of 1:400), anti-Cytochrome C, 136F3 (dilution of 1:200), anti-cleaved caspase-3, Asp175 (dilution of 1:400), anti-Smac/Diablo, D553R (dilution of 1:200) and anti- OPA1, D7C1A (dilution of 1:400) overnight at 4 °C. Then, the slides were washed 3 times each lasting 10 min in PBS and then incubated with secondary antibodies (ab150077 from Abcam, Cambridge Biomedical Campus, Cambridge, UK) for 2 h at room temperature in the dark. DAPI,#C0065 from Solarbio Life Sciences, Beijing, China (1:100PBS) was added for 5 min and poured off, washed in PBS for 3 times each lasting 5 min, dried, the anti-quenching agent was added(#P0126, Beyotime Biotechnology, Shanghai, China), glass slips were fixed on the slides, and samples were stored at −20 °C for 2 h and taken for microscopic imaging. The tissue images were captured using a confocal fluorescence microscope (Ni-U942877, Nikon, Tokyo, Japan), and the images representing the proteins in the motor cortex were then quantified using FIJI-ImageJ as described in [33].

### 4.7. Statistical Analysis

GraphPad Prism version 8.0.0 for windows (GraphPad Software, San Diego, CA, USA) was used for all analyses. One-way ANOVA followed by Dunnett’s post hoc tests, respectively, were used to measure statistical significance. Results are presented as mean ± SEM, and *p* < 0.05 was considered statistically significant.

## 5. Conclusions

This study demonstrates that swimming exercise ameliorated motor activity, memory, and learning associated with HIE by suppressing mitochondrial apoptosis via the Cyto.C/cleaved caspase-3 and AIF signaling pathways. Additionally, swimming exercise intervention has been shown to stabilize the mitochondrial cristae and membrane potential as depicted by the reversal of Smac/Diablo and OPA1 in rats with HIE. As a result, this is an attractive and viable foundation for additional research to decipher more molecular pathways that could be manipulated to effectively manage HIE and the resultant deficits.

While this study successfully demonstrated the role of exercise in reversing the structural and functional deficits in the mitochondria caused by HIE, it did not exhaust all the possible methods for establishing how the mitochondrial cristae are stabilized; this is critical for future research on this subject.

## Data Availability

Not applicable.

## Abbreviations

AIF	Apoptosis-inducing factor
Cyto. C	Cytochrome C
Endo G	Endonuclease G
HIE	Hypoxic ischemic encephalopathy
HIE, NT	Hypoxia ischemic encephalopathy with no exercise training
HIE, T	Hypoxia ischemic encephalopathy with exercise training
MFI	Mean fluorescence intensity
MWM	MWM Morris water maze
SHAM, NT	Sham group with no exercise training
SHAM, T	Sham group with exercise training
Smac	Second mitochondria-derived activator of caspase
OMM	Outer mitochondrial membrane
OPA1	Optic atrophy-1
